# Tensile Properties of the Murine Ventral Vertical Midline Incision

**DOI:** 10.1371/journal.pone.0024212

**Published:** 2011-09-07

**Authors:** Mark A. Carlson, Dennis Chakkalakal

**Affiliations:** 1 Department of Surgery, University of Nebraska Medical Center, Omaha, Nebraska, United States of America; 2 Surgery 112, Omaha Veterans Affairs Medical Center, Omaha, Nebraska, United States of America; 3 Orthopedic Research Laboratory, Omaha Veterans Affairs Medical Center, Omaha, Nebraska, United States of America; 4 Department of Surgery, Creighton University Medical Center, Omaha, Nebraska, United States of America; Harvard Medical School, United States of America

## Abstract

**Background:**

In clinical surgery, the vertical midline abdominal incision is popular but associated with healing failures. A murine model of the ventral vertical midline incision was developed in order to study the healing of this incision type.

**Methodology/Principal Findings:**

The strength of the wild type murine ventral abdominal wall in the midline was contained within the dermis; the linea alba made a negligible contribution. Unwounded abdominal wall had a downward trend (nonsignificant) in maximal tension between 12 and 29 weeks of age. The incision attained 50% of its final strength by postoperative day 40. The maximal tension of the ventral vertical midline incision was nearly that of unwounded abdominal wall by postwounding day 60; there was no difference in unwounded vs. wounded maximal tension at postwounding day 120.

**Conclusions/Significance:**

After 120 days of healing, the ventral vertical midline incision in the wild type mouse was not significantly different from age-matched nonwounded controls. About half of the final incisional strength was attained after 6 weeks of healing. The significance of this work was to establish the kinetics of wild type incisional healing in a model for which numerous genotypes and genetic tools would be available for subsequent study.

## Introduction

The healing of skin and aponeurotic incisions has high relevance for clinical surgery, especially with regard to the kinetics of incisional strength gain and the final incisional strength attained [1], [2]. In general surgery, the commonly-utilized vertical midline incision (through the linea alba) has been associated with dehiscence and hernia rates of ∼1% and 5–10%, respectively [1], [3]; decades of research (not reviewed here) has been devoted to minimizing these complications. Studies on the effects of various cytokines, genes, drugs, physical factors, etc. on incisional healing typically have focused on one or two time points early in the healing process. In contrast, there are few studies (see Discussion) which document rate of strength gain and plateau of final strength in an incisional wound, particularly in the vertical midline incision that is relevant to general surgery. In the present study, we determined the kinetics of strength gain and plateau of final strength in the ventral vertical midline incision in mice. The mouse model was chosen because numerous genotypes and genetic tools are available for this species that could be employed in future studies of incisional healing.

## Results

**Figure 1:**
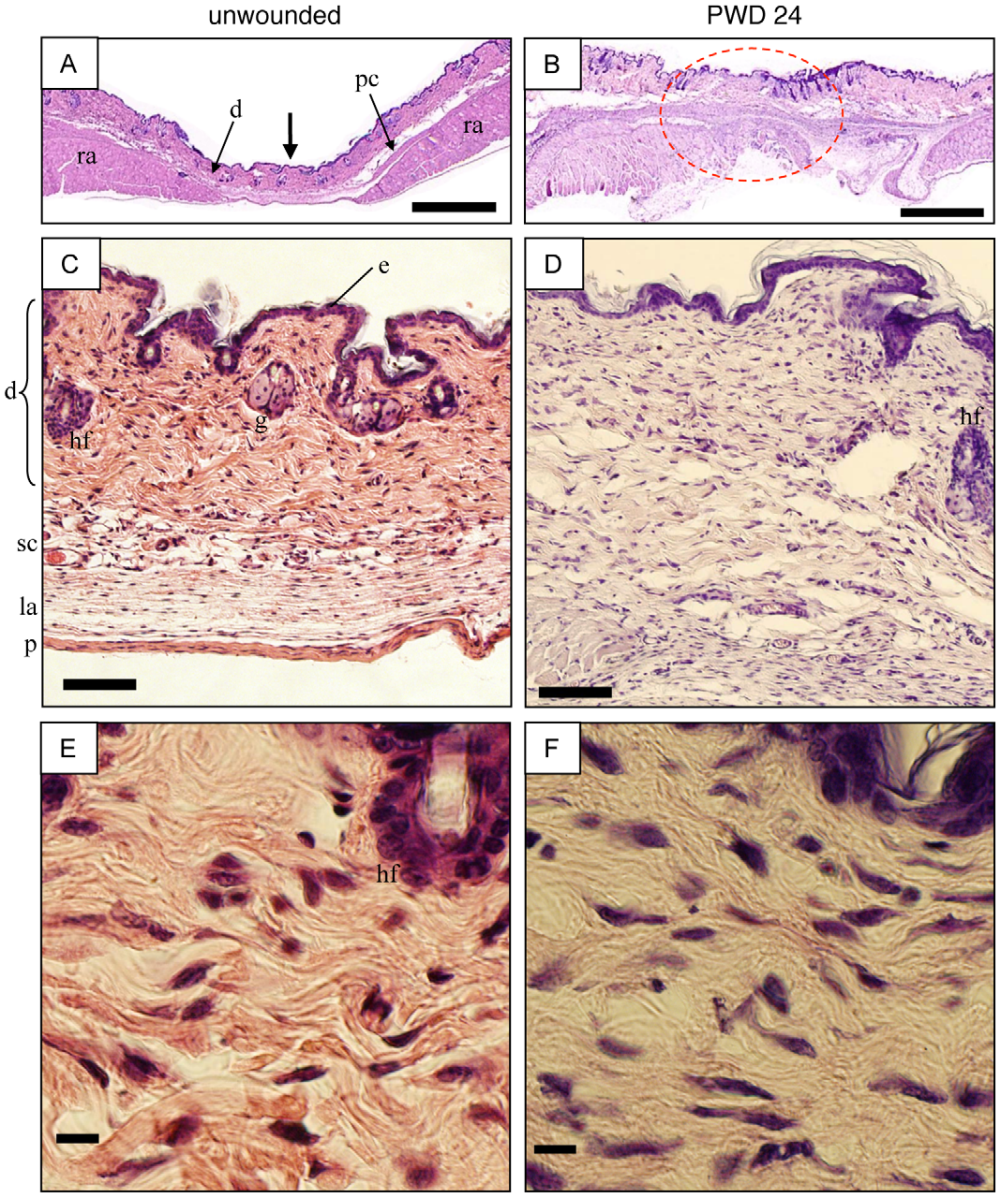
Gross and microscopic anatomy. Murine ventral abdominal wall from unwounded (A, C, E) *vs.* wounded (B, D, F) 16 week old subjects is shown. (A&B) Transverse whole section of the ventral midline abdominal wall stained with hematoxylin and eosin. Heavy black arrow = site of vertical midline incision (red line in Figure 3A); dotted oval = site of incisional wound. Bars = 1 mm. (C&D) Low-power micrograph of abdominal wall corresponding to the above heavy black arrow and dotted oval. Bars = 100 µm. (E&F) Higher magnification of ventral midline abdominal wall at the sub-epidermal level. Bars = 10 µm. d = dermis; e = epidermis; g = gland; hf = hair follicle; la = linea alba; p = peritoneum; pc = panniculus carnosus muscle; ra = rectus abdominus muscle; sc = subcutaneous tissue.

The microscopic anatomy of transverse sections of the wounded murine abdominal wall in the region of the linea alba is compared with that of the nonwounded animal in Figure 1 (panels B, D, F *vs.* A, C, E, respectively). The vertical midline incision in these transverse sections cut through the central portion of the section (heavy black arrow in Figure 1A). At this location, the abdominal wall consisted of epidermis, dermis, subcutaneous tissue, the linea alba, and peritoneum (Figure 1C). Lateral from the midline, the abdominal wall became thickened by the presence of muscular layers (rectus abdominus and panniculus carnosus; Figure 1A).

The time elapsed between surgery and euthanasia was defined as “postwounding day” (PWD). On PWD 24, the incision was occupied by a typical wound matrix (Figure 1B, D, F) that was free of appendages such as hair follicles and glands (the one hair follicle present in Figure 1D is at the dermal margin). At higher magnification (Figure 1E, F), the cells in the wounded dermis may have been more elongate and had a higher population density compared to the cells in the nonwounded dermis, but this and other putative differences were subtle. The thickness of the abdominal wall at the site of the incision was about twice greater in the wounded *vs.* the unwounded subject (Table 1).

**Table 1:** Ultimate tensile strength (UTS) of the mouse abdominal wall on PWD 24 compared with UTS of abdominal wall from uninjured, age-matched, controls at 16 weeks of age.

strip type	strip width, W (mm)	strip thickness, T (mm)	strip area, W×T (mm^2^)	UTS (MPa)
uninjured	5.0	0.457±0.022	2.28	4.6
PWD 24	5.0	0.986±0.092	4.93	0.59

Width of the test strip (Figure 3) before testing was measured with a ruler. Thickness of the strip before testing was measured at the midline, using ImageJ software on digital micrographs (6–8 per mean) of wound sections (http://rsb.info.nih.gov/ij/). UTS was calculated by dividing the mean maximal tension by the mean strip area.

1 MPa = 10^6^ Newton/m^2^.

**Figure 2:**
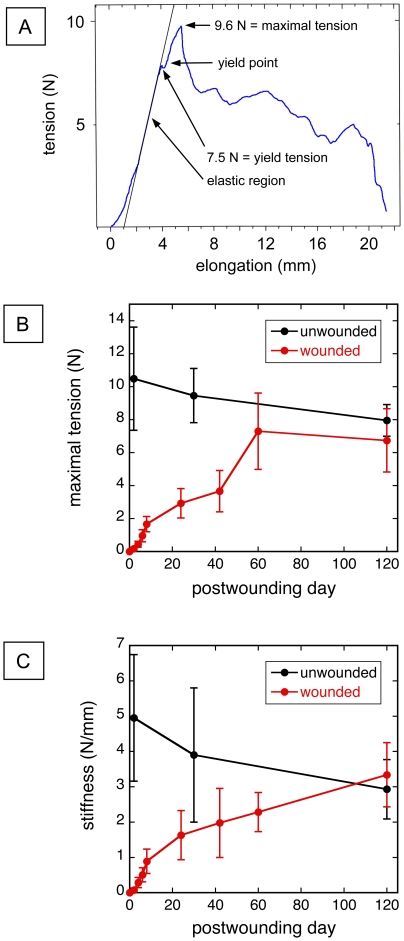
Tensile properties of wounded *vs.* unwounded abdominal wall. (A) Typical plot of tensile force vs. elongation in the disruption of a single test strip (blue curve) generated during tension testing of a test strip from uninjured abdominal wall loaded, at the elongation rate of 2 mm/min. The black straight line is the calculated slope of the curve in the elastic region. (B) Maximal tension sustained by tissue test strips *vs.* postwounding day (PWD) of the injured mouse abdominal wall compared with maximal tension *vs.* age of uninjured abdominal wall from age-matched control mice. Each data point represents the mean maximal tension (± SD) of 14–24 test strips. (C) Tensile stiffness *vs.* PWD for the same specimens as in panel B. Tensile stiffness was calculated as the slope of the straight line in Figure 2A, and is a measure of the resistance of the tissue to elongation under the applied tensile force. This is not the modulus of elasticity, since the curve in Figure 2A is not a stress *vs.* strain curve.

A plot of tensile force *vs.* elongation of a test strip (nonwounded abdominal wall in this example) is shown in Figure 2A. \ The linear relationship between the force and elongation in the elastic region is represented by the straight line superimposed on the curve. This test strip underwent irreversible plastic deformation of approximately 0.5 mm (yield-point elongation) at a yield force of ∼7.5 N (see Methods for further discussion of definitions). The test specimen then appears to undergo a change, reminiscent of “strain hardening” in metals, indicated by a small linear elastic region and a small nonlinear elastic region, each corresponding to approximately 0.5 mm elongation, before reaching the maximum tensile force of 9.6 N.

The changes in maximum tensile force sustained by the wounded abdominal wall as a function of postwounding day is compared with that of the nonwounded abdominal wall of age-matched mice in Figure 2B. The nonwounded abdominal wall specimens were tested at 12, 16, and 29 weeks of age of the mice (corresponding to PWD 0, 30, and 120 in the injured mice). The mean maximum tension of the nonwounded abdominal wall declined gradually with the age of the mouse: 9%, 7% and 11% in the three consecutive time intervals; but this trend had only marginal significance (p = 0.053, ANOVA). The decline in the mean maximum tension of the wounded abdominal wall from day 60 to 120 was 9%, which is consistent with the age-related decline of maximum tension of the nonwounded abdominal wall during the same period. On day 120, the mean maximum tension of the wounded abdominal wall was 16% less than that of the nonwounded abdominal wall, but this difference was nonsignificant (p = 0.084).

The recovery of tensile stiffness (i.e., the resistance to elongation in the elastic region of the curve in Figure 2A) of the wounded abdominal wall and the age-related decline in stiffness of the nonwounded abdominal wall (Figure 2C) have the general characteristics of the corresponding data on maximal tension (Figure 2B). The age-related decline in mean stiffness of the nonwounded abdominal wall during 12 to 29 weeks of age is approximately 40% (Figure 2C), compared with a 24% decline in mean maximal tension (Figure 2B). During this time the stiffness of the wounded abdominal wall completely recovered to the level of the nonwounded controls.

The ultimate tensile strength (UTS; see Methods) of the wounded abdominal wall on PWD 24 (0.6 MPa) was almost an order of magnitude less than that of the nonwounded tissue of age-matched controls (16 weeks), as shown in Table 1. Cross-sectional area data was available only for test strips from 16 week-old control animals and from wounded animals on PWD 24 (Table 1). Therefore, statistical comparison of UTS of wounded vs. nonwounded tissue at all time points was not possible.

## Discussion

1. The study of the kinetics of tensile strength in healing incisions is an essential component of experimental surgery, particularly if dealing with vascular or gastrointestinal anastomosis, tendon repair, hernia repair, and so forth. This type of experimentation has high clinical relevance, because failure of incised and sutured tissue, whether arterial wall, intestinal wall, aponeurosis, etc. has remained a vexing problem in clinical surgery [1], [4]. As a soft indication of this relevance, a Google Scholar search spanning 2008–2010 using the key words “incisional healing,” “anastomosis healing” or “tendon healing” returned 16,700, 7,710, and 15,000 hits, respectively.

2. Unfortunately, research into the kinetics of tensile strength recovery in sutured wounds has to be modeled in animals, because such studies in humans are not feasible and/or ethical. Available studies of tensile strength of fascia in humans typically has been limited to study of specimens harvested from cadavers [5], [6], [7], [8], [9], which has made construction of a kinetic curve for human incisional healing (such as in Figure 2B) essentially impossible. So in the present study we chose to model the ventral vertical midline incision, which is well-known for its propensity to fail, as manifested acutely by dehiscence [1] or chronically by incisional hernia [10].

3. Clinically, the important mechanical property determined for each test strip in this study is the maximal tension that the test specimen can handle (see Figure 2A); this tension also is known as the “disruption force” of the specimen[1]. If this level of tension is exceeded in a surgical wound, then wound dehiscence or incisional hernia is a likely outcome. In animal and cadaver studies of incisional wound healing [5], [6], [11], [12], [13], [14], [15], [16], [17], [18], [19], [20], [21], [22], [23], [24], [25], [26], [27], this disruption force (a property of the test specimen) typically is reported. The reporting of the UTS (a property of the material, irrespective of specimen size) typically has not been done in studies of tissue tensile strength. The lack of UTS reporting makes it difficult to compare results among different tensiometric studies.

4. The strength of the healing murine abdominal wall at day 120 in our study seemed to approach the strength of the age-matched uninjured abdominal wall (Figure 2B), but the strengths of both groups also appeared to be suffering from age-related decline. Previous work on the maximal force sustained by various biological tissues (including skin, bone, and tendon) in human and animal models has noted a general age-related decline in strength [28], [29], [30], [31], [32], [33], [34], [35].

5. The rapidity at which an incision gains strength and the ultimate strength that the incision attains have high clinical relevance [1], [2]. A sutured incision that gains strength at relatively slow rate and/or has a relatively low final strength will be at a higher risk for failure (e.g., dehiscence or hernia) compared to an incision which gains strength more quickly and/or has a higher final strength. A convenient method to estimate rapidity of strength gain is the time required for an incision to gain 50% of its final strength (the “t_50_” value) ; in the present study this time can estimated from the plot in Figure 2B as ∼40 days.

6. Derivation of the t_50_ value requires enough experimental groups tested at appropriate times such that a plot shown in Figure 2B can be constructed. The results of previous studies which contain enough of this data are summarized in Table 2. These studies were identified with (1) a Google Scholar search using combinations of the following keywords: fascia, aponeurosis, linea alba, skin, dermis, incision, tensile, tensiometric, wound, disruption, strength; and (2) a manual search through the bibliographies of the papers found with Google Scholar. Given the time period that was searchable (nearly 100 years), the actual number of studies identified for Table 2 was not that large (n = 12).

**Table 2:** Gain of tensile strength after incision and suture of various tissue (previous literature).

Citation	Species	Tissue incised	time to 50% strength (day)	final strength (% of intact tissue)
Howes *et al.*, 1929 [47]	dog	Skin	9	na
Howes *et al.*, 1929 [47]	dog	rectus sheath	6	na
Botsford, 1941 [48]	guinea pig	skin, paravertebral	7	na
Hartzell *et al.*, 1942 [49]	guinea pig	skin, ventral midline	7	na
Fast *et al.*, 1947 [50]	rabbit	rectus sheath	3*	21
Nelson *et al.*, 1951 [51]	rabbit	rectus sheath	7	80
Douglas, 1952 [52]	rabbit	aponeurosis, lumbar	30	70
Levenson *et al.*, 1965 [22]	rat	skin, paravertebral	30	85
Adamsons *et al.*, 1970 [53]	rabbit	skin, ventral	40	40
Lichtenstein *et al.*, 1970 [54]	rabbit	rectus sheath	13	40
Forrester *et al.*, 1970 [55]	rat	skin, paravertebral	55	67
White *et al.*, 1971 [56]	guinea pig	skin, paravertebral	180	85

The values of “time to 50% strength” (relative to final wounded strength) and “final strength” (relative to intact tissue) were interpolated from plots of strength *vs.* time in the original manuscripts, and therefore are approximate values.

*Strength measured with sutures *in situ*.

7. Interestingly, values for “t_50_” in Table 2 were widely divergent for both skin (7–180 days) and fascia (3–30 days). Fascia was demonstrated in one direct comparative study to gain strength quicker than skin [36]. The wide range of t_50_ values in Table 2 was somewhat troublesome, and is not easily explained. Possible causes for such divergent t_50_ values might include: species-specific differences in healing; location-specific differences in healing (i.e., dorsal skin *vs.* ventral skin); differences in the tensile testing methodology (these studies spanned a 40 year period); or inadequate numbers and/or time points of test groups.

8. Another observation from Table 2 is that full recovery of native strength in surgical incisions typically does not occur. Final strengths were in the range of 20–85% of the nonwounded tissue. Statistical testing of the Table 2 data was not available, however, so a firm conclusion on differences in final strength was not possible. Similar to the t_50_ values, the divergent final strengths are difficult to explain. Interestingly, one study of equine linea alba [37] demonstrated that the tensile strength of incised and sutured tissue actually exceeded that of nonwounded tissue after 24 weeks of healing (there was no statistical difference at the 8 week time point). We were not able to find corroborative studies that indicated that final strength of incised/sutured tissue could exceed that of native tissue.

9. In our study, the tensile strength of the abdominal wall was measured in the ventral vertical midline incision (i.e., through the linea alba), which is a common incision type utilized in open abdominal surgery. Our intent was to model this incision in a genetically “pliable” animal, with the intent that future studies could take advantage of the wide selection of genetically-modified mice and also the molecular tools that are available for that species. Interestingly, we found that the isolated linea alba in mice did not contribute a measurable degree of strength to the nonwounded abdominal wall. The tensile strength resided in the skin. In any event, we documented the rate of strength gain and the plateau strength for this incision in mice, and it is this data in this incision for this species which sets this study apart from previous studies.

10. The data of the present study focused on the tensile strength of skin strips of constant width and length. As mentioned above, the UTS needs to be determined in order to compare the native strength of one material with another. In our study, the UTS was ∼5 MPa for nonwounded skin, while the wounded skin at 24 days was almost an order of magnitude less (Table 1). For comparison, the UTS of spider silk is 1–2 GPa [38], various formulations of steel are in the range of 600–1,000 MPa [39], bone is ∼150 MPa [40], tendon is ∼50–100 MPa [41], and polypropylene is ∼30 MPa [42].

11. The relevance of the data in this report is that a commonly-utilized incision in abdominal surgery has been modeled in the mouse, which has not previously been done. This modeling should engender future study of the healing of this incision in this animal model, for which there are numerous genetic variants and related tools available for research use. Improvement and optimization of midline incisional healing would be relevant to open abdominal surgery, because (1) the need for open abdominal surgery will not go away in the near future [43]; (2) the midline incision frequently is used during open abdominal surgery [44]; and (3) the midline incision has a well-documented incidence of failure [10].

12. In summary, the strength of the murine ventral vertical midline incision at postoperative day 120 was not statistically different from nonwounded abdominal; ∼50% of final incisional strength was present at 6 weeks. These findings should have implications for future studies of the effect of various interventions on incisional healing, in that evaluation of tensile strength at an early time point (e.g., 7 or 14 days, as typically has been done) may have limited clinical relevance, because relatively little tensile strength returns in the first two weeks. If an investigator desires to evaluate an interventional effect on tensile strength at a single time point then, based on our data, the experiment may be more relevant if the investigator chooses 6 weeks as the time point, since differences in effect should be easier to discriminate at this interval.

## Materials and Methods

### Ethics Statement

The use of mice was approved by the Institutional Animal Care and Utilization Committee (IACUC) of the Omaha Veterans Affairs Medical Center (approval ID number 00308).

### Surgical Procedure

C57BL/6J mice (male, 12 weeks old at time of wounding) were anesthetized with inhalational isoflurane. Hair on the ventral abdomen was clipped, followed by depilation with a calcium hydroxide agent (Nair®); and the skin then was scrubbed with a soap solution containing 0.5% triclosan (5-chloro-2-(2,4-dichlorophenoxy)phenol; CV Medicated Lotion Soap, Steris Corp.) A 2.0 cm full-thickness abdominal wall incision (exposing the viscera) was made in the ventral midline (i.e., through the linea alba), beginning just below the xiphoid process and extending inferiorly. The incision was immediately closed using a running 7-0 polypropylene suture (Prolene, Ethicon) with 3.5× loupe magnification, taking all layers of the abdominal wall (epidermis to peritoneum). The stitch interval was 1 mm, and the bite size was 1 mm; therefore, each incision contained ∼20 suture loops. No dressing was applied, and no antibiotics were used.

### Preparation of Tension Test Specimens

**Figure 3:**
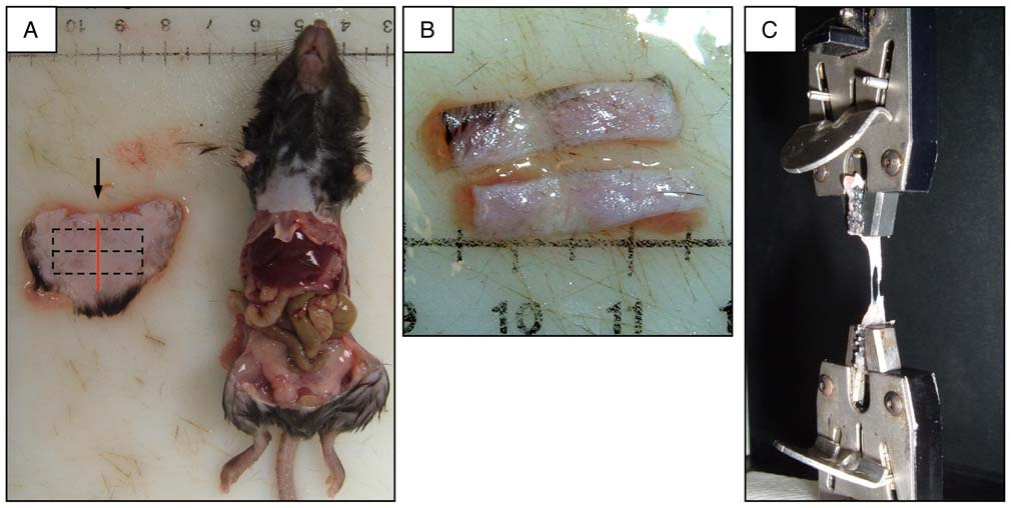
Wound disruption assay. (A) Ventral image of supine subject after harvest of ventral abdominal wall; note exposure of abdominal viscera. Excised specimen is shown to the left in its approximate anatomic position. Dashed lines demonstrate how test strips were cut from the specimen. Vertical red line shows location of vertical midline incision (scale = cm). (B) Test strips cut from abdominal wall specimen similar to that shown in panel A; orientation is same as that shown in panel A (scale = cm). (C) Disruption of a test strip in the tension tester. This specimen is nearly in two halves, connected by two fine strands of tissue.

On the tensile testing day, each animal underwent AMVA-approved CO_2_ euthanasia [45]. All suture material was removed. The anterior abdominal wall was excised with all layers from the costal margins superiorly, out to both extreme flanks laterally, and down to the hindquarters inferiorly (Figure 3A). This specimen contained the linea alba, which bisected the specimen in the vertical direction (red line in Figure 3A). A 20 mm by 10 mm rectangle of abdominal wall was excised from the central portion of this specimen (Figure 3A), and then cut into two test strips, each 20 mm by 5 mm (Figure 3B). The site of incision was positioned in the middle of the test strip during subsequent tensile testing (Figure 3C). The test strips of each subject were inked to discriminate the superior from inferior strip. The strips then were placed in phosphate-buffered saline (PBS) until testing, which was always done within 1 hr of euthanasia.

### Tension Testing: Definitions

We used a standard tension testing procedure [46] to generate a curve of tensile force *vs.* elongation (Figure 2A), and to determine the yield force, maximum tensile force, and the stiffness of wounded *vs.* unwounded skin strips. In this report, “tension” and “tensile force” are used interchangeably.

The initial part of the curve in Figure 2A was a small nonlinear region usually observed in tensile testing of soft tissue specimens, which is devoid of meaningful data. This was followed by the elastic (i.e. linear) region that culminates at the yield point, at which plastic deformation occurred (that is, an increase in elongation without an increase in force). For soft tissue specimens such as a skin strip, the yield point usually occurs before the peak force is reached.

The tension at the yield point was defined as the *yield force*, and was 7.5 N in Figure 2A. The peak force, or maximum tensile force, that the strip withstood in Figure 2A was 9.6 N. If the composition of the test material is homogeneous, then the *ultimate tensile strength* (UTS) of the material (or simply “tensile strength”) is defined as the ratio of the peak force to the cross-sectional area of the specimen at test initiation. The typical unit of UTS is the pascal (1 Pa = 1 N/m^2^).

In this report, the strip composition was assumed to be homogeneous for the determination of the UTS. The UTS is an intrinsic property of the material, and is determined both by material composition and test conditions. The UTS should not be influenced by the size of the test sample.

The peak force (measured in newtons) is related, but technically not the same, as the UTS (measured in pascals); but since care was utilized in this study to maintain a reasonably constant strip size, we employed peak force as a “surrogate” or approximation for UTS in the plots of Figure 2 and in the discussion of wound kinetics.

The *stiffness* of the test specimen was defined as the slope of the elastic (linear) region of the force vs. elongation curve (Figure 2A) that preceded the yield point. Stiffness is a measure of the resistance of the test specimen to elongation, and is expressed in N/m.

Stiffness is related but different from the *modulus of elasticity*, which is defined as the slope of the stress *vs.* strain curve in the linear elastic region, with units of Pa. Similar to the UTS, the modulus of elasticity is an intrinsic property of the material. And analogous to the utilization of peak force as a surrogate for UTS, stiffness was used as a surrogate for the modulus of elasticity.

### Tension Testing: Technique

Tension testing was performed with an Instron® Model 1011 Tester, with Series IX Instron® software, a 5 kg load cell, and 4 mm spring-loaded tissue grips (Figure 3C). Initial focus was placed on tensile testing of the linea alba, since this has been the clinically relevant layer in vertical midline incisions [1]. It was observed, however, that the peak force recorded from isolated unwounded linea alba in the murine model was below the level of detection (<0.1 N) of the tension tester. Most, if not all, of the tensile strength of the murine abdominal wall at the ventral midline was sustained by the dermis (data not shown). Therefore, it was decided that subsequent tensile testing of the ventral midline abdominal wall would be done on all layers at this location (i.e., epidermis to peritoneum), with no attempt to isolate individual layers.

Preliminary experimentation with a variety of grip surfaces demonstrated that the least tissue slippage occurred when the grip jaws were covered with coarse-grit wet sandpaper (glued onto the jaws with cyanoacrylate). All tension tests were performed with this sandpaper-grip modification; the sandpaper was changed every ∼10 tension tests. Each test strip was loaded into the grips such that ∼5 mm of the central portion of the test strip (containing the linea alba) was exposed and centered between the superior and inferior grip at the test initiation (Figure 3). The rate of displacement was constant at 2 mm/min. The test strips were kept wet with PBS before and during the test run. The tensile force was applied to each test strip, elongating it beyond the maximal tensile force until the residual tension was less than 0.1 N, which occurred after complete or near-complete disruption of the test strip (Figure 3C).

Other preliminary tension tests revealed that excessive tissue slippage occurred within the grip jaws when the lateral musculature (i.e., the rectus and panniculus) was left intact on the test strips (data not shown). If this musculature was dissected off the test strips prior to testing, then minimal or no slippage occurred. Dissection and removal of the lateral muscles from the test strips did not disturb the integrity of the aforementioned layers at the midline. Therefore, all subsequent tension tests were performed on test strips that had the lateral musculature removed. Preliminary experimentation with the rate of displacement yielded an optimal rate of 2 mm/min. Faster rates were associated with an unacceptable decrease in experimental precision (data not shown).

Statistical analysis of the data (expressed as mean ± standard deviation) was performed using ANOVA and the two-tailed unpaired t-test with unequal variances; statistical significance was set at p<0.05.
